# Effect of treatment with dental space maintainers after the early extraction of the second primary molar: a systematic review

**DOI:** 10.1093/ejo/cjad006

**Published:** 2023-03-30

**Authors:** Tuka Tabatabai, Heidrun Kjellberg

**Affiliations:** Smile Tandvård, Landskrona, Skåne,Sweden; Specialist Clinic of Orthodontics, Public Dental Service, Skåne, Lund, Sweden

## Abstract

**Background:**

Early loss of second primary molars may lead to different malocclusions, mainly caused by the mesial migration of the first permanent molar. To prevent space loss in the dental arch, different types of space maintainers (SM) are used.

**Objectives:**

The main objective of this systematic review is to examine the evidence in the literature regarding the effect of SM, including the clinical effect, risk of developing caries and periodontal disease, patient satisfaction, and cost-effectiveness after the premature loss of the second primary molar in children.

**Search methods:**

The present systematic review was made according to PRISMA. The literature search was performed using four databases (last search 30/8/2022): PubMed, Cochrane Central Register of Controlled Trials (CENTRAL), Scopus, and Web of Science.

**Selection criteria:**

The studies included were randomized controlled trials, economic evaluations, and non-randomized clinical studies with a defined control group.

**Data collection and analysis:**

Data collected by the two authors were in regard to reports, studies, participants, research designs, and interventions. The assessment of the risk of bias was made using the ROBINSON-I tool.

**Results:**

The search yielded 1058 articles after the removal of duplicates. Two studies were included in the final review with a moderate risk of bias, and measured space changes in the dental arch and the periodontal status of patients treated with SM. The main results indicate that treatment with SM can preserve arch length, but also cause an increase in plaque accumulation and other periodontal parameters. However, there is an overall lack of scientific evidence regarding the effect of the treatment.

**Limitations:**

No studies that fulfilled the eligibility criteria were found on cost-effectiveness, risk of caries development, and patient satisfaction.

**Conclusions:**

The scientific evidence is lacking regarding the clinical effect, cost effect, and side-effects such as caries and periodontal disease when using SM in children with a premature loss of the second primary molar.

**Registration:**

PROSPERO Registration (CRD 42021290130).

## Introduction

Early loss of primary teeth, because of tooth decay or trauma, can affect the primary dentition, the permanent dentition, or both (1,2). It can cause crowding that may affect a child’s self-esteem and quality of life (1) and lead to changes in the dental arch such as ectopic eruption of permanent teeth and other malocclusions (1,3). Today, crowding is the most common problem seen by orthodontists, and dentists and patients need guidance on how to intervene to prevent them (1).

The greatest dimensional alterations have been seen after the loss of the second primary molars, which have mainly been attributed to the mesial migration of the first permanent molar (2,4,5). To prevent this space loss, interceptive treatment with different types of space maintainers (SMs) has been used (6–8). Given their frequent use in paediatric dentistry after the early loss of primary molars (5), it is important to understand the clinical evidence and costs associated with SM, as well as the patient’s own experience (9).

Earlier studies have also investigated interceptive treatment with SM regarding the potential side effects of periodontal health and the presence of caries (6–8). However, a considerable variety in the treatment methods, eligibility criteria, study designs, and research approaches has resulted in outcomes and conclusions that can be conflicting and may sometimes be difficult to interpret and compare. Therefore, an overview of the present knowledge seems important. Earlier literature reviews have investigated the effect of SM in children (9–12). However, there are no systematic reviews evaluating the use of SM compared with no treatment after the premature loss of the second primary molar.

## Objectives

The objective of this systematic review was to assess and evaluate, in a structured and evidence-based manner, the existing scientific evidence regarding the clinical effects, side effects, patient satisfaction, and cost effects of interceptive treatment with SM compared with no treatment after the premature loss of the second primary molar in children.

## Method

The present systematic review was conducted using the criteria of the Preferred Reporting Items for Systematic Reviews and Meta-Analyses (PRISMA) (13). The study protocol was registered at PROSPERO (Registration number CRD 42021290130).

### Protocol and eligibility criteria

The problem specification and the criteria for the final search were developed according to the PICO strategy (Table 1). The studies included were randomized controlled trials, prospective studies, economic evaluations, and non-randomized clinical studies with a defined control group. No publication year restriction was used, and the studies were written in English or Swedish and had available abstracts. No follow-up limitation was used during the search.

**Table 1.** Inclusion criteria according to PICO.

Population	Patients (age 4–15 years) with premature loss of the second primary molar and with an erupted first permanent molar
Intervention	Different types of dental space maintainers
Comparator	No space maintainers
Outcomes	- Clinical effect (space loss, angulation of teeth or other reported effects on the dentition) - Presence of caries (DMFT/DMFS or other cariological parameters) - Presence of periodontal disease (plaque index, gingival index, bleeding on probing, bone resorption or other periodontal parameters) - Patient satisfaction (oral health-related quality of life (OHRQoL) measures or any other measure to assess patient expectation, experience, and satisfaction) - Cost effectiveness (total costs, total effectiveness, incremental cost effectiveness ratio (ICER), or any other measure to assess cost effectiveness)

### Literature search

The literature search was performed using four databases (last search 30/8/2022; no data restriction used): PubMed, Cochrane Central Register of Controlled Trials (CENTRAL), Scopus, and Web of Science. The databases were searched with the following keywords: ((((((((((space maintainers) OR space maintainer) OR space maintenance) OR (Band and loop)) OR (Crown and loop)) OR Nance palatal arch) OR Lower lingual arch) OR Resin space maintainers) OR bonded space maintainer) AND orthodontics). Additionally, a review of the reference lists of the relevant included articles was manually performed, and articles with relevant titles were obtained. A further search for grey literature was made in opengrey.eu.

### Extraction and interpretation of data

The extraction and interpretation of data were completed by both authors individually and information was collected regarding each report (author, year of publication, country), study (study design, sample characteristics), the participants (indication of the use of SM, type of dentition, e.g. primary or mixed dentition), the research design and features (sampling mechanism, treatment assignment mechanism, follow up period, dropouts, complications) and the intervention (type of SM, clinical effect, cost effect, periodontal health, caries, patient satisfaction).

In case of ambiguity, the consensus was achieved through a discussion between the two authors. Both authors independently analysed the retrieved titles and abstracts according to the defined eligibility criteria. If the methodology in a particular article was unclear, the full text was further analysed. The duplicates retrieved from the literature search were then excluded. Also, a hand search was made by a manual screening of the titles in the reference lists in the finally chosen articles.

### Risk of bias of individual studies

The evaluation of the risk of bias was performed using the ROBINS-I tool (Risk Of Bias In Non-randomized Studies-of Interventions) and the studies included could be determined as ‘low’, ‘moderate’, ‘serious’, ‘critical’, or ‘unclear’ risk of bias (11). The articles were evaluated individually by both authors. If different opinions occurred, they were discussed until a common evaluation was determined.

### Risk of bias across studies

The risk of bias across studies would have been considered if the methodology was comparable across studies.

### Summary of results and statistical analysis

A meta-analysis would have been performed if there was a homogeneity in the study designs and treatments. Relevant data of interest were collected and organized into tables to present the study and patient characteristics of the studies included as well as the effects of SM.

## Results

### Literature search

**Figure 1.**
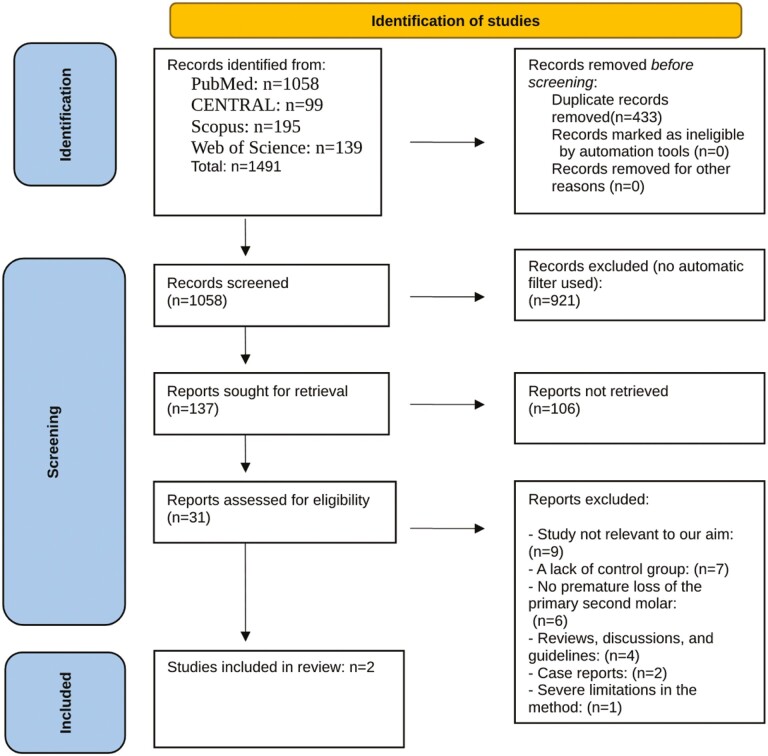
Flow diagram of the literature search.

Of the total 1491 articles that were retrieved after the electronic searches, 137 relevant abstracts were retrieved, of which 31 were further analysed by reading the full text. Two articles were considered relevant according to our eligibility criteria; see Figure 1.

The search in CENTRAL, Scopus, Web of Science, and the search of reference lists and grey literature did not result in additional articles other than the articles included from Pubmed.

### Outcome

The data is summarized in Table 2. Both studies were clinical studies with a prospective nature; one was made in Jordan, and the other one in Turkey. The studies had the same treatment modalities regarding the treatment with different types of SM compared with a control group. In one of the studies, the control group consisted of teeth surfaces without SM in the same patients treated with SM, and in the other study, the control group consisted of a separate group of patients without SM (6,7). Ethical approval was given for both studies and was provided respectively by the JUST Institutional Research Board and Ankara University’s ethics committee (6,7).

**Table 2.** Summarized results.

Article	Study design	Material (size, age, drop outs)	Follow-up	Type of SM	Method/measurements	Results
Arikan et al., 2015	Observational cohort study	40 patients, 20 boys, 18 girls, 2 drop outs _______ Age: 4–10 years	- T0: baseline, the insertion of SM - T1: 1 month - T2: 3 months - T3: 6 months	Band and loop and removable space maintainers with an acrylic base	A clinical examination to measure plaque index, gingival index, bleeding on probing index	- Plaque index: In group 1 (fixed SM) plaque index increased in regions with SM. No significant difference in plaque index in group 2 (removable SM) compared to regions without SM - Gingival index: In group 1 plaque index increased in regions with SM No significant difference in group 2 in removable SM versus no SM - BoP: BoP increased in regions with SM (group 1) in T0–T2 and T0–T3, while no significant difference T0–T1 in group 2 compared to regions without SM *- E. faecalis* counts increased over T0 were observed for patients using removable and fixed space maintainers
Owais et al., 2010	A prospective clinical study, a quasi-randomized trial	67 patients, 39 males, 28 females, 9 drop outs _______ Age: approximately 10 years	Up to 2.42 ± 0.63 years	- Group 1: Lower lingual arch of 0.9 mm SS wire - Group 2: lower lingual arch of 1.25 mm SS wire - Control group: without SM	Measurements of arch length, arch depth, inter-canine width, inter-molar width, lower second primary molar extraction space using lateral cephalograms, dental pantomograms, and study casts	- Increased lower incisor inclination in both groups with SM - Significant increase in lower molar angulation in patients without SM

However, different outcomes were measured. Space conditions were measured by studying the arch length, extraction space using lateral cephalograms, and study casts (7). The periodontal status was measured with regard to plaque index, gingival index, and bleeding on probing (6).

The authors concluded that the use of different space maintainers led overall to an increase in the periodontal index parameters and the number of microorganisms and plaque index in the oral cavity (6). Regarding the space changes, it was shown that lower molar angulation to the mandibular plane increased in all groups, but significance was achieved only in patients without SM, meanwhile, the lower incisor inclination to the mandibular plane increased in patients with SM after the extraction of the primary teeth (7).

Neither of the studies included or reported information regarding the prevention of malocclusions, patient satisfaction, cost-effectiveness, or the presence of caries.

### Risk of bias within studies

The overall risk of bias was measured as ‘moderate’ in both studies (6,7), see Table 3. Both studies included statistical analyses and both studies had a small sample size and did not describe the recruitment of patients. In one study, no power analysis was made, and the groups were divided by an alternation of odd and even numbers; thus, the study was assessed to be a quasi-randomized trial (7). Arikan et al.’s was classified as a cohort study or an observational study (6). The randomization process in this study was not mentioned (6).

**Table 3.** Risk of bias according to ROBINS-I tool.

Study	Confounding	Selection bias	Classification of intervention	Intended intervention	Missing data	Measurements of outcomes	Reported results	Overall
Arikan et al. (4).	Moderate	Moderate	Low	Low	Low	Moderate	Moderate	Moderate
Owais et al. (5).	Moderate	Moderate	Low	Moderate	Moderate	Moderate	Moderate	Moderate

The eligibility criteria were mentioned in both studies and the combination of lateral cephalograms, dental pantomograms, and study casts to measure alterations in the dentition and space were evaluated as relevant methods (7). Also, a clinical examination including plaque index according to Silness and Loe (6), gingival index, and bleeding on probing index to measure gingival health were considered as acceptable.

Drop-out rates were disclosed in both studies. However, in one of the studies, no further data were given regarding the patient data (e.g., age, gender, etc.) in the dropouts or how the lack of follow-up was distributed between the different groups (7).

### Risk of bias across studies and meta-analysis

Due to the variety in the methodology and study design, no risk of bias across studies or meta-analysis could be performed.

## Discussion

### Summary of evidence

This systematic review summarized the current evidence regarding the effect of space maintainers after the premature loss of the second primary molar. Two studies were included in this review with a moderate risk of bias (6,7). The studies measured the clinical effectiveness including space loss, and periodontal health (6,7). Even though there are several studies measuring the effect of SM in general, many of them did not have a control group without SM, and/or did not study the effect of SM after the premature loss of the primary second molar.

‘The main findings of the studies included in this review are that treatment with SM seemed to preserve arch length, and at the same time increase the inclination of the lower incisors to the mandibular plane (7). Also, the lower molar angulation in patients without SMs was significantly increased (7). The treatment also caused an increase in plaque accumulation in groups treated with fixed SMs compared to patients without SMs (6). No studies that fulfilled the eligibility criteria were found on cost-effectiveness, caries, and patient satisfaction.’

### Clinical effectiveness

Today, available resources within the health sector (personal, time, facilities, equipment, and knowledge) are limited (14). Hence, failure to analyse the economic aspects of dental health services may lead to unsustainable over-expenditure or a reduction of resources in other areas of healthcare (15). The studies included in our report did not examine cost-effectiveness, patient satisfaction, or the long-term benefit of using SM.

Hence, today, there is a lack of sufficient evidence as a basis for dental healthcare providers to use SM in children with a premature loss of the second primary molar. In this decision, the survival rate, and possible complications of the treatment with SM, including cement failure, band breakage, solder breakage, wire breakage, and the loss of the appliance, should be considered (7). Owais et al. investigated the correlation of complications in groups with a lower lingual holding arch device (LLHA) with two different gauges (0.9 and 1.25 mm) of stainless steel (SS). The results showed that patients with 1.25 mm LLHA had more problems than patients that received treatment with 0.9 mm LLHA regarding cement failures, band breakages, and solder breakages, which they explained by the stiffness of the 1.25 mm SS wire (7).

In both treatment groups, the proclination of the lower incisors was increased relative to the A-Pog line. These results are like some earlier findings which show that arch perimeter loss can be reduced, but at the expense of the proclination on mandibular incisors in patients treated with a lower lingual arch (16). However, contradicting results with backward tipping of lower incisors are also found (17). In a study investigating the effect of a lower lingual arch in 23 children, a backward tipping of the lower incisors by 0.51 degrees was observed during a follow-up period of 18 months (17). The angulation of the lower first permanent molar as a result of the LLHA has also been investigated, and a distal tipping was found in all groups, a result that is in agreement with findings reported by others (16,18).

Overall, the results from Owais et al*.,* presented in this review showed that both groups treated with SM preserved arch length throughout the study duration (7). These results contradict an earlier study from Alnahwi et al., measuring space loss following the premature loss of primary second molars, where the space loss in the groups with SM and without SM was similar (18). A possible explanation for the different results is that space loss in these studies was measured differently. Owais et al. included lateral cephalograms, dental pantomograms, and study cast in the method, and SMs were inserted followed by the extraction of the primary second molar (7). Meanwhile, Alnahwi et al. measured space loss using bitewing and periapical radiographs with no information regarding the calibration of the pictures and in most cases SMs were placed within 2 months after the extraction and, in the case of 10 teeth, SMs were placed between 1 and 2 years after the extraction. According to Macena et al., the major space changes in the dental arches occur during the first 3 months after the extraction of the deciduous molars, indicating that SM should be applied immediately after extraction (4). Tunison et al. (12) also highlight the impact of individual occlusal characteristics on space loss. Besides the location of the primary tooth, it has been shown that space loss is greater in the mandible compared to the maxilla, when tooth loss occurs earlier in age and in crowded compared to spaced dentitions (12).

Another possible side effect of SM is increased eruption difficulties of the second permanent molar (19). This effect was not mentioned in the studies included in our report.

### Periodontal disease and caries

Previous studies have shown that there may be a correlation between the use of orthodontic appliances and the retention of plaque and the development of gingivitis (20). Arikan et al. examined changes in the microflora and parameters including plaque index, bleeding index, pocket depth, and the presence of *E*. *faecalis* after the use of SM (6). It was concluded that both fixed and removable SM can cause an increase in plaque accumulation. Children with fixed appliances showed an increase in plaque and bleeding index compared to patients with removable SM, and the authors suggested that special attention should therefore be given to young patients with fixed appliances. Other studies have shown similar results regarding periodontal parameters such as bleeding and probing and pocket depth, and the periodontal and microbiologic parameters with orthodontic bands compared with a control group, when investigating removable and fixed orthodontic appliances (21,22). However, periodontal parameters such as bleeding and probing, plaque accumulation, and gingivitis can be seen as temporary and reversible symptoms due to poor oral hygiene (20). Severe conditions also include a loss of the marginal bone (20). This was not examined in the included studies.

Difficulties in maintaining good oral hygiene, and the increase of plaque accumulation, may contribute to the demineralization of enamel surfaces in patients with fixed orthodontic bands (20). Even though the plaque accumulation was measured as higher in patients with SM, the potential effect for the development of caries was not investigated (6). Caries are the most common reason for early extractions of primary teeth (23). Patients treated with SM may therefore have a history of caries. Previous caries experience is the single strongest factor for the prediction of future caries (24). The potential risk for caries’ development in patients treated with SM is therefore important to study in the future.

### Strengths and limitations

This systematic literature review was conducted according to PRISMA. This model fulfils the criteria for repeatability and minimizes the risk of the conclusions being affected by chance or arbitrariness. The results in the studies included in this review show a clear variation in study design and measured variables that made a meta-analysis impossible to perform. During the search, several studies were excluded since they lacked a control group without SM. Both studies included in this review had a small sample size and did not describe the recruitment of patients. In one of the studies, no power analysis was made (6). Given the fact that a small sample size was used in the study and no power analysis was made, there is a risk of low power, and insignificant outcomes may be achieved even though clear differences may occur. Other limitations were the moderate quality of the studies included and the lack of studies in the fields of patient satisfaction, caries, and cost-effectiveness.

### Future research

More and better prospective clinical trials and randomized-controlled trials with sufficient sample sizes and control groups are required to determine the effect of the treatment after premature extractions of primary second molars. Future research should also include an analysis of the cost and side effects of the treatment as well as patient satisfaction.

## Conclusion

The available evidence in this study shows that treatment with SM may preserve arch length, but patients treated with SM also showed an increase of plaque accumulation and some other periodontal parameters. However, these outcomes should be very cautiously interpreted, due to the methodological limitations of the studies included.

Overall, there is a lack of evidence in the literature regarding the clinical effectiveness, cost-effectiveness, and side effects such as caries and periodontal disease when using SM. Hence, today, there is a lack of sufficient scientific evidence as a basis for dental healthcare providers to use SM in children with a premature loss of the second primary molar (25).

## Data Availability

I have read the journal’s requirements for reporting the data underlying my submission (data policy in EJO Author instructions) and have included a Data Availability Statement within the manuscript.
